# Intravenous Immunoglobulins Promote an Expansion of Monocytic Myeloid-Derived Suppressor Cells (MDSC) in CVID Patients

**DOI:** 10.1007/s10875-022-01277-7

**Published:** 2022-04-29

**Authors:** Miriam Simón-Fuentes, Silvia Sánchez-Ramón, Lidia Fernández-Paredes, Bárbara Alonso, Kissy Guevara-Hoyer, Miguel A. Vega, Angel L. Corbí, Ángeles Domínguez-Soto

**Affiliations:** 1 Myeloid Cell Laboratory, Centro de Investigaciones Biológicas, CSIC, Ramiro de Maeztu, 9, 28040 Madrid, Spain; 2 Hospital Universitario Clínico San Carlos, IML and IdSSC, Madrid, Spain

**Keywords:** Intravenous immunoglobulins, Common variable immunodeficiency disorders (CVID), Human monocytes, Monocytic myeloid-derived suppressor cells

## Abstract

Common variable immunodeficiency disorders (CVID), the most common primary immune deficiency, includes heterogeneous syndromes characterized by hypogammaglobulinemia and impaired antibody responses. CVID patients frequently suffer from recurrent infections and inflammatory conditions. Currently, immunoglobulin replacement therapy (IgRT) is the first-line treatment to prevent infections and aminorate immune alterations in CVID patients. Intravenous Immunoglobulin (IVIg), a preparation of highly purified poly-specific IgG, is used for treatment of immunodeficiencies as well as for autoimmune and inflammatory disorders, as IVIg exerts immunoregulatory and anti-inflammatory actions on innate and adaptive immune cells. To determine the mechanism of action of IVIg in CVID in vivo, we determined the effect of IVIg infusion on the transcriptome of peripheral blood mononuclear cells from CVID patients, and found that peripheral blood monocytes are primary targets of IVIg in vivo, and that IVIg triggers the acquisition of an anti-inflammatory gene profile in human monocytes. Moreover, IVIg altered the relative proportions of peripheral blood monocyte subsets and enhanced the proportion of CD14^+^ cells with a transcriptional, phenotypic, and functional profile that resembles that of monocytic myeloid-derived suppressor cells (MDSC). Therefore, our results indicate that CD14 + MDSC-like cells might contribute to the immunoregulatory effects of IVIg in CVID and other inflammatory disorders.

**Supplementary Information:**

The online version contains supplementary material available at 10.1007/s10875-022-01277-7.

## Introduction

Common variable immunodeficiency disorders (CVID) are one of the most common clinically important primary immune deficiencies. CVID encompasses a group of heterogeneous primary antibody failure syndromes characterized by hypogammaglobulinemia associated with reduced or absent specific antibody production [1, 2]. CVID patients commonly suffer from recurrent infections of the gastrointestinal and upper respiratory tracts [3, 4], and inflammatory conditions and autoimmunity are also frequent in CVID patients [4–6]. Immune dysregulation in CVID might be secondary to defects of B-cell differentiation and function, chronic and reduced T cell function, and altered NK cells and dendritic cells function, with chronic microbial translocation possibly contributing to the systemic immune activation and altered homeostasis of lymphocytic and myeloid lineages [7–9]. Currently, immunoglobulin replacement therapy (IgRT), either intravenous (IVIg) or subcutaneous (SCIg), is a first-line therapy to prevent infections and aminorate all these immune alteration in CVID patients [10, 11].

IVIg is a preparation of highly purified polyclonal poly-specific IgG isolated from plasma of thousands of healthy donors and widely used for the treatment of primary and secondary immunodeficiencies, as well as autoimmune and inflammatory disorders (reviewed in [12]). Besides their antibody replacement effect, IVIg exerts immunoregulatory and anti-inflammatory actions on innate and adaptive immune cells [13–17], and various non-mutually exclusive mechanisms have been proposed [17–21] to explain its clinical effectiveness [18–24]. The high number of donors used to prepare IVIg is particularly relevant to its anti-inflammatory effects, given the extensive pool of unique IgG antibody repertoire and anti-carbohydrate repertoire [25]. The ample anti-inflammatory activity of IVIg has recently attracted attention to its potential therapeutic use for COVID-19 [26–29], COVID-19-related Kawasaki disease [30–32], and for the thrombotic thrombocytopenia observed after ChAdOx1 nCov-19 vaccination [33–35]. We have previously demonstrated that IVIg skews macrophage polarization through FcγR-dependent mechanisms [36] and that IVIg promotes tolerance towards inflammatory stimuli [37]. However, extrapolation of the prophylactically administration in animal models of disease falls short in providing definitive answers about its mode of action in humans in vivo, where IVIg is commonly used in numerous other therapeutic strategies [21].

To address the mechanisms of action of IVIg in vivo, we have now determined the phenotypic, transcriptomic, and functional profile of peripheral blood mononuclear cells (PBMC) from CVID patients prior and after IVIg infusion. Our results indicate that IVIg triggers the acquisition of an anti-inflammatory profile in PBMCs and monocytes, reduces the number of inflammatory circulating monocytes, and enhances the proportion of CD14^+^ monocytes whose phenotype and suppressor activity is compatible with that of myeloid-derived suppressor cells (MDSC). Our results indicate that monocytes are primary targets for the anti-inflammatory and inmmunosupressive effects of IVIg in CVID patients in vivo.

## Materials and Methods

### Patients and Clinical Samples

We studied a cohort of 11 CVID patients (age range of 20–70 years; mean age: 48.3 ± 13.8 years) followed at the Department of Clinical Immunology at the Hospital Clínico San Carlos (Madrid, Spain). CVID patients were diagnosed according to the classification of European Society of Immune Deficiencies (ESID) and the Pan-American Group for Immune Deficiency (PAGID) [38, 39]. Given the inherent heterogeneity in CVID manifestations, we took into account the classification of clinical phenotypes proposed by Chapel et al. [40], and our cohort included patients with “no-disease-related” complications (“infections only” phenotype) (*n* = 5) and patients with inflammatory/autoimmune/lymphoproliferative complications (“inflammatory” phenotype) (*n* = 6) (Supplementary Table 1). The study protocol was approved by the Ethics Committee of Hospital Clínico San Carlos (Madrid, Spain), and all subjects provided signed informed consent (Project 19/284-E).

All CVID patients were in a stable state with no apparent acute infection, and received IgRT as part of their routine treatment. The mean cumulative monthly dosage of IgRT was 400 mg/kg, with an infusion time from 4 to 6 h established according to the individual patient´s tolerability. The mean IVIg dose administered at the time of blood sampling was 28.7 ± 3.9 g. None of the 11 patients were taking steroids or other immunosuppressive or immunomodulatory drugs at the time of the study or the previous 6 months. Blood was obtained from CVID patients both before and after (6 h) receiving IgRT infusion, and PBMC were isolated over a Lymphoprep (Nycomed Pharma, Oslo, Norway) gradient according to standard procedures. Monocytes, T lymphocytes, and B lymphocytes were purified from PBMC by magnetic cell sorting using CD14, CD3, and CD20 immunomagnetic beads (Miltenyi Biotech, Bergisch Gladbach, Germany), respectively.

### Microarray Analysis

Global gene expression analysis was performed on RNA obtained from PBMC, monocytes, T lymphocytes, and B lymphocytes isolated immediately before or after IVIg therapy of four independent patients. RNA isolation, microarray analysis (whole human genome microarray, Agilent Technologies, Palo Alto, CA), and statistical treatment of microarray data were performed following previously described procedures [41–43]. Microarray data were deposited in the Gene Expression Omnibus (http://www.ncbi.nlm.nih.gov/geo/) under accession nos. GSE133835 (PBMCs), GSE133907 (CD14 cells), GSE158576 (CD20 cells), and GSE158573 (CD3 cells). For Gene Set Enrichment Analysis (GSEA) (http://software.broadinstitute.org/gsea/index.jsp) [44], the gene sets available at the website, as well as previously defined gene sets, were used.

### Phenotypic Analysis of Monocyte

Whole blood samples were collected before and after IVIg administration. Immediately after collection, blood sample was incubated at room temperature for 20 min with the indicated fluorescently tagged monoclonal antibodies. Following RBC lysis (RT, 15 min) using FACS Lysing solution (Becton Dickinson), cells were washed twice and analyzed on a FACS Canto II (Becton Dickinson) flow cytometer. For simultaneous surface and intracellular staining, cell surface antigen staining was performed first, and cells were later resuspended in Buffer Perm/Wash 1 × solution, treated with fixation and permeabilization solution (4 °C, 30 min in the dark) and subjected to intracellular staining. Monocyte subpopulations were phenotypically identified by a 8-color flow cytometry single platform assay using anti-CD14-APC Cy7, CD16-FITC, CX3CR1-PerCP Cy5, HLA-DR-BV510, CD86-PE, CCR5-BV421, CCR2-APC, and TNF-PE mAbs (BD, Becton- Dickinson Biosciences, Franklin Lakes, NJ).

### T Cell Suppression Assay and Cytokine Secretion

Human peripheral blood CD4^+^ lymphocytes were isolated from CVID patients using magnetic cell sorting with anti-CD4 microbeads (Miltenyi Biotec), resuspended in RMPI 5% human AB serum (Sigma-Aldrich), and added into flatbottom 96-well plates (10^5^ cells/well) that had been coated overnight with anti-human CD3 (10 μg/ml, BD Biosciences) and anti-human CD28 (1 μg/ml, BD Biosciences). Then, CD14^+^ cells isolated from CVID patients (both before and after IVIg infusion) were resuspended in RMPI 5% human AB serum, and co-cultured with CD4^+^ lymphocytes at the indicated ratios. After 48 h, [^3^H]thymidine was added (1 uCi/well, Perkin Elmer) during the last 20 h of coculture and thymidine incorporation was determined using a MicroBeta2 2450 Microplate Counter. Cell culture supernatants from the suppression assay were collected after 48 h and IFN-γ levels determined by ELISA (PBL Assay Science) following the protocol supplied by the manufacturers.

### Statistical Analysis

Unless otherwise indicated and for comparisons of means, statistical analysis was performed using the Student *t* test, and a *p* value < 0.05 was considered significant (*p < 0.05; **p < 0.01; ***p < 0.001).

## Results

### In Vivo IVIg Infusion Prompts the Acquisition of an Anti-inflammatory and Immunosuppressive Transcriptional Profile in Peripheral Blood Mononuclear Cells from CVID Patients

**Fig. 1:**
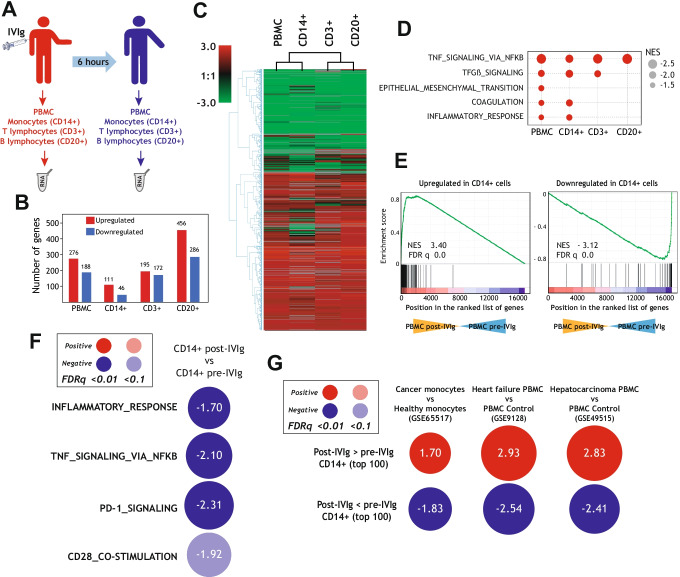
Transcriptional changes induced by IVIg infusion in peripheral blood monocytes, T lymphocytes, and B lymphocytes from CVID patients. **A** Schematic representation of the transcriptional analysis performed on PBMCs, monocytes, T, and B lymphocytes isolated from CVID patients before and after IVIg infusion. **B** Gene expression changes (pval < 0.001 and |log2fold change|> 1) in PBMC (GSE133835), CD14^+^ (GSE133907), CD3^+^ (GSE158573), and CD20^+^ (GSE158576) cells from CVID patients before and after IVIg infusion. **C** Non-supervised hierarchical clustering analysis of the IVIg-regulated gene expression in PBMC, monocytes (CD14^+^), T lymphocytes (CD3^+^), and B lymphocytes (CD20^+^). **D** Summary of the GSEA results on the ranked comparison of IVIg-treated vs. untreated cell subsets isolated from CVID patients, and using the Hallmark gene set (h.all.v7.4.symbols.gmt) from the GSEA webpage (http://software.broadinstitute.org/gsea/index.jsp) [44]. Normalized Enrichment Scores (NES) are indicated. **E** GSEA of IVIg-regulated genes in CD14^+^ cells (Upregulated or Downregulated; pval < 0.001 and |log2fold|> 1) on the ranked comparison of the transcriptome of PBMCs from CVID patients before (PBMC pre-IVIg) and after IVIg infusion (PBMC post-IVIg). Normalized enrichment score (NES) and false discovery rate (FDR q-val) are indicated in each case. **F** Summary of GSEA with the Hallmark and Reactome gene sets (h.all.v7.4.symbols.gmt, c2.cp.reactome.v7.4.symbols.gmt) from the GSEA webpage (http://software.broadinstitute.org/gsea/index.jsp) on the ranked comparison of the transcriptomes of CD14^+^ monocytes isolated from CVID patients after (CD14^+^ post IVIg) and before (CD14^+^ pre-IVIg) IVIg infusion. The color of the circles illustrates the type and statistical significance (FDRq value) of the enrichment of each comparison (positive, red; negative, blue). The area of each circle is proportional to the normalized enrichment score of each comparison, which is also indicated. **G** Summary of GSEA with the gene sets containing the Top 100 genes Upregulated [”Post-IVIg > pre-IVIg CD14^+^ (top 100)”] or Downregulated [”Post-IVIg < pre-IVIg CD14^+^ (top 100)”] in CD14^+^ post-IVIg relative to CD14^+^ pre-IVIg, and on the ranked comparison of the indicated transcriptomes, retrieved from GEO GSE65517 [45], GSE9128 [46] and GSE49515 [47]. The color of the circles illustrates the type of enrichment of each comparison (positive, red; negative, blue). The area of each circle is proportional to the normalized enrichment score of each comparison, which is also indicated

To identify the cell types that mediate the anti-inflammatory and immunosuppressive effects of IVIg in vivo, we initially compared the transcriptional profiles of PBMCs, CD14^+^ monocytes, CD3^+^ T cells and CD20^+^ B cells isolated from CVID patients before and 6 h after IVIg infusion (Fig. 1A). IVIg triggered substantial changes in the gene signature of all analyzed cell subsets (Fig. 1B) and, in fact, modulated the expression of a common set of genes in CD14^+^, CD3^+^, and CD20^+^ cells (Fig. 1C). Gene ontology analysis using GSEA further supported the similarity among the transcriptional effects of IVIg on PBMC and the three cell types, with a shared negative enrichment of genes associated to the term “TNF signaling via NFkB” (Fig. 1D). Moreover, GSEA showed a marked resemblance of the gene ontology terms negatively enriched in IVIg-treated CD14^+^ monocytes and IVIg-treated PBMCs, including the terms “Coagulation” and “Inflammatory Response” (Fig. 1D). Indeed, the genes significantly upregulated or downregulated by IVIg in CD14^+^ monocytes were found to be positively or negatively enriched in the transcriptome of PBMC post-IVIg (Fig. 1E). As a whole, these analysis revealed the overlapping transcriptional effects of IVIg on PBMCs and CD14^+^ monocytes in vivo, and that the IVIg-induced transcriptional changes in PBMCs and CD14^+^ monocytes are compatible with the anti-inflammatory and immunosuppressive effects of IVIg [17–21].

Based on the above findings, we next focused our subsequent analysis on the transcriptome of CD14^+^ monocytes after IVIg treatment. As shown in Fig. 1F, IVIg infusion provoked a very significant negative enrichment of the terms “TNF signaling via NFKB,” ´”inflammatory response,” "PD-1 signaling," and "CD28 co-stimulation" in IVIg-treated CD14^+^ cells (Fig. 1F). Furthermore, Enrichr and GSEA gene ontology analysis revealed that the transcriptional changes induced by IVIg in CD14^+^ monocytes greatly resemble the transcriptional changes that take place in monocytes in breast cancer patients (GSE65517) [45], and in PBMCs from heart failure (GSE9128) [46] and hepatocellular carcinoma [47] patients (GSE49515) (Fig. 1G). Specifically, the genes specifically upregulated (or downregulated) in IVIg-treated CD14 + monocytes were similarly upregulated (or downregulated) in monocytes or PMBCs from the indicated conditions (Fig. 1G), where immunosuppression predominates and profound phenotypic and functional changes take place in peripheral leukocytes [45–49]. In addition, a similar result was obtained when the expression of genes modulated by IVIg in PBMCs was analyzed (Supplementary Fig. 1A). Altogether, these results illustrate the ability of IVIg to impair the acquisition of an inflammatory and immuno-stimulatory gene signature in human monocytes in vivo, and that IVIg infusion prompts the acquisition of a transcriptome that resembles the gene profiles of peripheral blood myeloid cells found in disorders associated with strong immunosuppression.

### Phenotypic Consequences of IVIg Infusion on Peripheral Blood Monocytes from CVID Patients

**Fig. 2:**
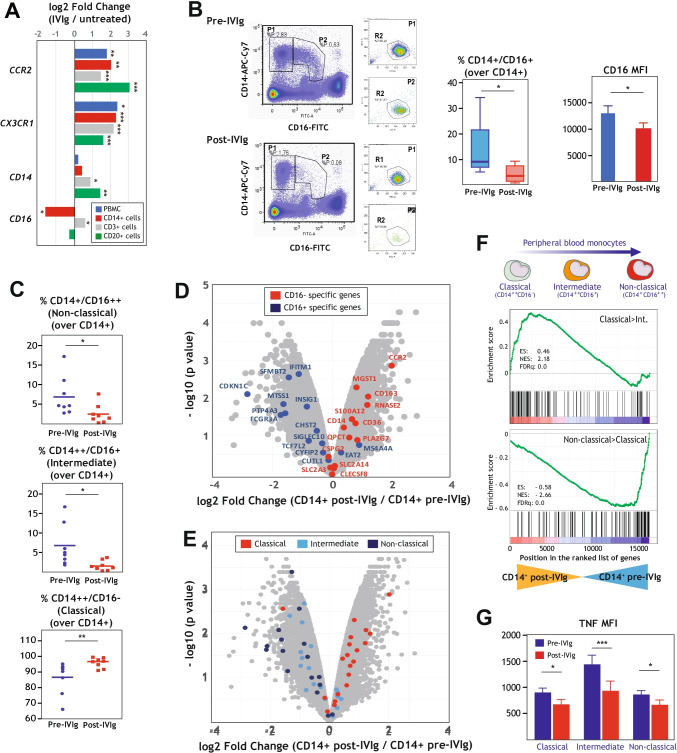
Transcriptional and phenotypic evidences for the IVIg-mediated alteration of the proportion of monocyte subsets in peripheral blood from CVID patients. **A** Expression levels of monocytic markers (*CCR2*, *CX3CR1*, *CD14*, and *CD16*) in different blood cell populations (PBMCs, CD14^+^ cells, CD20^+^ cells and CD3^+^ cells) before and after IVIg infusion (*p < 0.05; **p < 0.01; ***p < 0.001). **B** CD16 expression on the cell surface of monocytes before and after IVIg treatment in patients with CVID. The gating strategy for identification of monocyte subsets in the peripheral blood from CVID patients is indicated. Monocytes were gated by CD14 and CD16 expression and then, selected gates (P1 and P2) analyzed by FSC and SSC parameters (left panels). The percentage of CD16^+^/CD14^+^ cells in all CD14^+^ monocytes and CD16 mean expression for CD16^+^/CD14^+^ monocytes is shown. Mean ± SEM of 8 patients are shown (*p < 0.05; **p < 0.01) (right panels). **C** Percentage of classical (CD14^++^/CD16^−^), intermediate (CD14^++^CD16^+^), and non-classical monocytes (CD14^+^CD16^++^) before and after IVIg treatment (*p < 0.05; **p < 0.01). **D** Volcano plot of the comparison of the transcriptomes of “CD14^+^ post-IVIg” and “CD14^+^ pre-IVIg,” with indication of the genes that mark CD16^+^ monocytes (CD16^+^-specific genes, blue dots) and CD16^−^ monocytes (CD16^−^-specific genes, red dots) as reported in [52] (GSE16836). **E** Volcano plot of the comparison of the transcriptomes of “CD14^+^ post-IVIg” and “CD14^+^ pre-IVIg,” with indication of the genes that mark classical monocytes (red dots), intermediate monocytes (light blue dots) and non-classical monocytes (dark blue dots) as reported in [53]. **F** GSEA analysis of indicated gene sets (upper panel, genes with higher expression in classical than in intermediate monocytes [53]; lower panel, genes with higher expression in non-classical than in classical monocytes [53]) on the ranked comparison of the transcriptome of monocytes from CVID patients before (CD14^+^ pre-IVIg) and after IVIg infusion (CD14^+^ post-IVIg). Enrichment score (ES), normalized enrichment score (NES), and FDRq is indicated for each analysis. **G** Determination of TNF expression in the three monocyte subsets classical, intermediate, non-classical) of CVID patients before (pre-IVIg) and after IVIg administration (post-IVIg), as determined by flow cytometry. Mean ± SEM of three independent samples from CVID patients are shown (*p < 0.05, ***p < 0.001)

To functionally assess the effects of IVIg on CVID monocytes, and since the expression of the CD16-encoding *FCGR3* gene is significantly downregulated by IVIg (Fig. 2A), we initially checked the consequences of IVIg infusion on the phenotype of peripheral blood monocytes. Three major human peripheral blood monocyte subsets exist, whose defining phenotypes are CD14^++^CD16^−^ (classical monocytes), CD14^++^CD16^+^ (intermediate monocytes), and CD14^+/−^CD16^+^ (non-classical monocytes) [50]. Flow cytometry showed a drastic and significant reduction of CD16^+^ (intermediate and non-classical) monocytes (Fig. 2B, left panel), as well as a reduction of 22% in the CD16 cell surface expression (Fig. 2B, right panel), in peripheral blood from IVIg-treated CVID patients. Besides, the percentage of monocytes expressing CX3CR1, another specific marker for CD16^+^ monoytes [51], was also significantly reduced in the peripheral blood of IVIg-treated CVID patients (Supplementary Fig. 1B), suggesting that the decrease in CD16^+^ monocytes in IVIg-treated CVID patients reflects changes in monocyte subsets proportions and is not merely due to IVIg-mediated occupancy or internalization of CD16. As a whole, flow cytometry revealed that the percentage of intermediate and non-classical monocyte subsets decrease upon IVIg treatment in CVID patients, while the percentage of the classical monocyte subset is elevated after IVIg infusion in vivo (Fig. 2C). Furthermore, the effects of IVIg on the relative proportion of monocyte subsets in CVID patients were substantiated through the analysis of the genesets that define the three major monocyte subsets [52, 53]. Indeed, the most specific marker genes for CD16^+^ monocytes (GSE16836) [52] were found to be preferentially expressed by pre-IVIg CVID monocytes, whereas post-IVIg monocytes displayed enhanced expression of genes that characterize CD16^−^ monocytes (Fig. 2D). Likewise, classical monocyte-specific genes [53] were expressed at higher levels in post-IVIg CVID monocytes, while intermediate- and non-classical-specific genes [53] were preferentially expressed by pre-IVIg CVID monocytes (Fig. 2E), and similar results were seen upon evaluation of the expression of additional monocyte subset-specific gene sets in pre-IVIg and post-IVIg CVID monocytes [53] (Fig. 2F). Therefore, the combination of cytofluorimetric and transcriptomic experiments provided solid evidence for a reduction in the intermediate (CD14^++^CD16^+^) and non-classical (CD14^+^CD16^++^) peripheral blood monocyte subsets in IVIg-treated CVID patients, a result that fits with the transcriptional data and is in agreement with previous reports [54]. Noteworthy, similar results were obtained in all CVID patients regardless of their clinical CVID phenotype.

Finally, since CD16^+^ monocytes are increased in several inflammatory and autoimmune diseases [55] and possess a more mature phenotype [52], we explored whether IVIg also affected the in vivo expression of TNF in the distinct monocyte subsets. Cytometry analysis after intracellular staining revealed that IVIg infusion significantly reduced TNF expression in both CD16^+^ and CD16^−^ monocyte subsets, albeit the inhibitory action was more profound in the intermediate CD14^+^CD16^+^ monocyte population (Supplementary Fig. 1C and Fig. 2G). Thus, all monocyte populations from CVID patients appear to exhibit a reduced inflammatory profile after IVIg treatment, what might contribute to the immunomodulatory actions of IVIg in vivo.

### IVIg Infusion in CVID Patients Increases the Level of Immunosuppressive M-MDSC in Peripheral Blood

The above results indicated that IVIg limits the pro-inflammatory capacity of monocytes in CVID patients, but did not fully justify the net anti-inflammatory and immunosuppressive action of IVIg [21]. An initial hint of the anti-inflammatory/immunosuppressor effect of IVIg was suggested by the resemblance of the transcriptome of post-IVIg monocytes to the gene signature of monocytes and PBMCs from diseases where elevated levels of myeloid-derived suppressor cells (M-DSC) are found (cancer, cardiomyophathy) [45–49] (Fig. 1G). MDSC are a heterogeneous population of immature myeloid cells that expands in chronic and acute inflammation and in various cancer types [56, 57], and are functionally defined by their potent ability to suppress T cell activation [57–59] through various molecular mechanisms [56, 58, 60]. MDSC include two phenotypically distinct subsets: monocytic MDSC (M-MDSC), characterized by a Lin − CD11b + CD14 + CD15 − HLA-DR − /low phenotype, and polymorphonuclear MDSC (PMN-MDSC), with a Lin − CD11b + CD14 − CD15 + HLA-DR − or Lin − CD11b + CD14 − CD66b + phenotypic profile [61, 62].

**Fig. 3:**
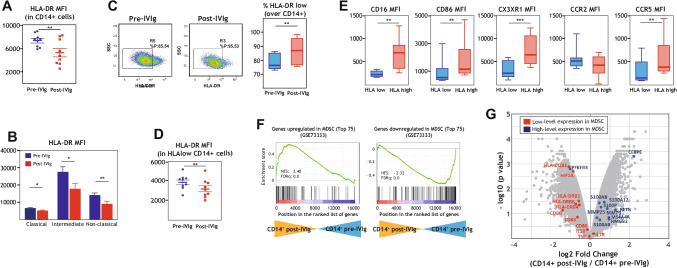
Peripheral blood monocytes from IVIg-treated CVID patients display MDSC-like phenotype and transcriptome. **A** Mean fluorescence intensity (MFI) of HLA expression in CD14^+^ monocytes from CVID patients before (pre-IVIg) and after IVIg infusion (post-IVIg), as determined by flow cytometry. Mean ± SEM of 9 independent CVID samples is shown (**p < 0.01). **B** Mean fluorescence intensity (MFI) of HLA expression in the three monocyte subsets (classical, intermediate, non-classical) from CVID patients before (pre-IVIg) and after IVIg infusion (post-IVIg), as determined by flow cytometry. Mean ± SEM of 9 independent CVID samples is shown (*p < 0.05; **p < 0.01). **C** Determination of the percentage of HLAlow CD14^+^ monocytes in peripheral blood from CVID patients before (pre-IVIg) and after IVIg infusion (Post-IVIg). A representative flow cytometry profile from a single CVID patient is shown in the left panels. Mean ± SEM of 9 independent CVID samples is shown (**p < 0.01) (right panel). **D** Mean fluorescence intensity (MFI) of HLA expression in HLAlow CD14^+^ cells from CVID patients before (pre-IVIg) and after IVIg infusion (post-IVIg), as determined by flow cytometry. Mean ± SEM of 9 independent CVID samples is shown (*p < 0.05; **p < 0.01). **E** Cell surface expression of M-MDSCs specific markers in HLAlow and HLAhigh monocytes from IVIg-treated CD14^+^ monocytes from CVID patients, as determined by flow cytometry. Mean ± SEM of eight independent CVID samples is shown (**p < 0.01; ***p < 0.001). **F** GSEA analysis of indicated gene sets (left panel, genes upregulated in MDSC, top 75; right panel, genes downregulated in MDSC, top 75), as reported in [63, 64] (GSE73333), on the ranked comparison of the transcriptome of monocytes from CVID patients before (CD14^+^ pre-IVIg) and after IVIg infusion (CD14^+^ post-IVIg). Normalized enrichment score (NES) and FDRq is indicated for each analysis. **G** Volcano plot of the comparison of the transcriptomes of “CD14^+^ post-IVIg” and “CD14^+^ pre-IVIg,” with indication of the genes highly expressed in MDSC (blue dots) or with low expression in MDSC (red dots), as indicated in [45, 65] (GSE65517)

To assess whether IVIg affects the level of MDSC, we initially compared HLA-DR expression, a hallmark of M-MDSCs [61], in CD14 + cells from CVID patients before and after IVIg infusion. HLA-DR expression was found to be significantly lower in post-IVIg CD14 + cells from CVID patients (Fig. 3A), a pattern that was observed in all monocyte subsets (Fig. 3B). Next, and following the consensus accepted for the identification of M-MDSCs [61], we determined the percentage of CD14 + HLA-DR^low^ cells before and after IVIg treatment and using the gating strategy shown in Supplementary Fig. 1D. Analysis of nine CVID patients revealed that IVIg treatment significantly increases the percentage of HLA-DR^low^ CD14 + cells (Fig. 3C), an increase that coincided with reduced cell surface expression of HLA-DR (Fig. 3D) and of the M-MDSCs markers CD16, CD86, CX3CR1 and CCR5 (Fig. 3E). This increase in the percentage of HLA-DR^low^ CD14 + cells was transitory, since a week after the infusion with IVIg only a few patients maintained certain levels of HLA-DR^low^ CD14 + cells increased compared to before IVIg treatment (data not shown). Therefore, IVIg infusion in CVID patients results in elevated proportion of CD14 + cells whose phenotype is compatible with that of M-MDSC. Further support for such an increase was obtained through GSEA on the transcriptomes of pre- and post-IVIg CD14^+^ cells. Specifically, the transcriptome of post-IVIg CD14^+^ cells was positively enriched in genes upregulated during in vitro MDSC induction (GSE73333) [63, 64], whereas pre-IVIg CD14^+^ showed a higher expression of genes whose expression is reduced in along monocyte-to-MDSC differentiation [63, 64] (Fig. 3F). Moreover, genes directly associated with MDSC proliferation and immunosuppressive function (GSE65517) [45, 65] were preferentially expressed by post-IVIg CD14^+^ cells (Fig. 3G). Therefore, these results indicate that IVIg treatment of CVID patients results in enhanced levels of CD14^+^ cells phenotypically and transcriptionally similar to M-MDSCs. Again, results were similar in all CVID patients regardless of their clinical CVID phenotype.

**Fig. 4:**
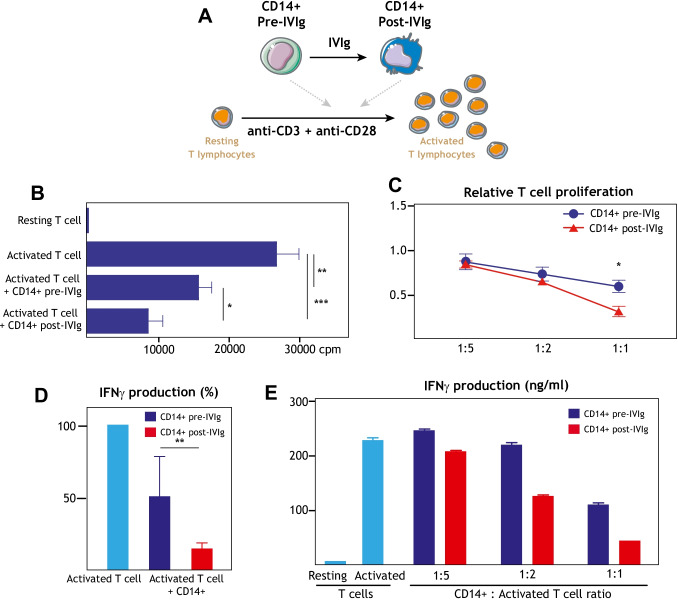
Peripheral blood monocytes from IVIg-treated CVID patients exhibit enhanced ability for suppression of T cell activation. **A** Schematic representation of the “T lymphocyte activation” suppression assay. **B** Determination of anti-CD3/anti-CD28-induced CD4^+^ T cell proliferation in the absence or presence (1:1) of CD14^+^ cells isolated from CVID patients before (CD14^+^ pre-IVIg) or after (CD14^+^ post-IVIg) IVIg infusion. Mean ± SEM of six independent experiments are shown (*p < 0.05; **p < 0.01; ***p < 0.001). **C** Relative anti-CD3/anti-CD28-induced CD4^+^ T cell proliferation in the presence of different ratios (CD14^+^:activated CD4^+^ ratio of 1:1, 1:2, 1:5) of CD14^+^ cells isolated from CVID patients before (CD14^+^ pre-IVIg) or after (CD14^+^ post-IVIg) IVIg infusion. Mean ± SEM of six independent experiments are shown (*p < 0.05). **D** Relative IFNγ production in the co-culture (1:1) of anti-CD3/anti-CD28-activated CD4^+^ T cells and CD14^+^ cells isolated from CVID patients before (CD14^+^ pre-IVIg) or after (CD14^+^ post-IVIg) IVIg infusion. Mean ± SEM of three independent experiments are shown (**p < 0.01). **E** IFNγ production in a representative co-culture of anti-CD3/anti-CD28-induced CD4^+^ T lymphocytes and CD14^+^ cells isolated from CVID patients before (CD14^+^ pre-IVIg) or after (CD14^+^ post-IVIg) IVIg infusion at different ratios (1:1, 1:2, 1:5). Mean ± SEM of three replicates of a single representative experiment is shown

Finally, to assess the inmunosuppressive ability of IVIg-induced M-MDSC-like CD14^+^ cells in CVID patients, they were co-cultured with autologous CD4^+^ T cells in the presence of T cell-activating anti-CD3 and anti-CD28 antibodies (Fig. 4A). As shown in Fig. 4B, activated CD4^+^ T cells cultured in the presence of post-IVIg CD14^+^ cells exhibited a weaker proliferation level than in the presence of pre-IVIg CD14^+^ cells, a feature consistent with the inmunosuppressive phenotype of M-MDSCs. As expected, a stronger inmunosuppressive effect was observed at lower CD4^+^ T cell/CD14^+^ ratios (Fig. 4C). More importantly, the presence of post-IVIg CD14^+^ cells also resulted in diminished production of IFNγ production (Fig. 4D), a feature that was also dependent on the CD4^+^ T cell/CD14^+^ ratio (Fig. 4E). Taken together, the transcriptional, phenotypic, and functional data indicate that IVIg infusion leads to higher levels of immunosuppressive M-MDSCs in CVID patients.

## Discussion

CVID is the most frequently diagnosed primary immunodeficiency. Baseline inflammatory complications, autoimmune diseases, and lymphoproliferation are common in CVID patients [4, 66–68], defining specific clinical phenotypes due to dysfunctional immune responses besides those seen upon recurrent infections [40]. Although the mechanisms underlying CVID-associated immune dysregulation remain largely unclear [68], previous reports have shown increased microbial translocation and systemic myeloid cell activation in CVID patients [69–72], whose chronic monocyte activation appears related to persistence of T cell activation and the inflammatory and lymphoproliferative complications [68, 73]. IVIg therapy is currently the treatment of choice for CVID, and we have previously shown that IVIg modifies the phenotype and function of myeloid cells in vitro and in vivo [36, 37]. Although it is accepted that the main therapeutic benefit of IVIg in CVID patients is the presence of pathogen-specific antibodies [74], IVIg-mediated cellular re-programming might also contribute to improve the control of infections in CVID patients [10, 11, 75]. We now report that IVIg treatment of CVID patients provokes the acquisition of an anti-inflammatory profile in PBMC and monocytes, and that IVIg enhances the percentage of CD14^+^ monocytes with a transcriptional, phenotypic, and functional profile compatible with those of myeloid-derived suppressor cells (MDSC). In parallel, IVIg infusion led to a marked reduction of the intermediate and non-classic monocyte subsets at the transcriptomic and phenotypic levels, a finding that is in agreement with the phenotypic effects of IVIg described by Cavaliere et al. in CVID patients [54]. Our results indicate that monocytes are preferential IVIg targets in vivo, and that the IVIg-mediated changes in the relative levels of monocyte subsets might contribute to the anti-inflammatory and immunosuppressive effects of IVIg in CVID. Of note, no difference was observed between CVID patients with either “infections only” or “inflammatory” phenotype. However, given the size of the analyzed cohort, these results warrant further validation in an independent and larger cohort.

Compared to healthy individuals, CVID patients have been recently found to exhibit higher levels of low-density neutrophils, whose phenotype and suppressive activity is consistent with granulocytic MDSC [70] that might contribute to the immune dysregulation in CVID. To our knowledge, the present report provides the first evidence for an IVIg-mediated increase in blood M-MDSC in CVID patients in vivo. We have also analyzed the levels of PMN-MDSC (CD15 + CD11b + CD33 + HLA-DRlow) in IVIg-treated CVID patients, but the results were inconclusive, and a larger cohort of CVID patients is required to clarify this issue in future studies. Our findings are in line with a previous report describing an increase in CD33^+^/CD11b^+^/HLA-DR^−^ MDSC in immune thrombocytopenia (ITP) patients treated with both IVIg and dexamethasone after 6 days [76], and also agree with an enhancement of CD33^+^/CD11b^+^/HLA-DR^−^ cells in spleen cells from ITP patients exposed in vitro to IVIg for 90 h [77]. Our results, however, indicate that IVIg enhances monocytic MDSC levels in peripheral blood as soon as 6 h after infusion, indicating an acute effect. MDSC appear to originate mainly from an emergency myelopoiesis [78], and their enrichment might be due to monocyte reprogramming into an immunosuppressive state, early release of bone marrow immature myeloid cells into the circulation (emergency myelopoiesis), or a combination of both mechanisms [79]. Whether the IVIg-induced increase in monocytic MDSC in CVID reflects a re-programming of peripheral blood monocytic cells or is secondary to release of bone marrow progenitors has yet to be addressed. Although the latter cannot be ruled out, since IVIg-treated CVID patients do not show elevated monocyte counts, and considering that IVIg re-program monocytes and macrophages in vitro [36, 37], it is reasonable to assume that IVIg directly shapes peripheral blood monocytes at the transcriptional and phenotypic level in vivo in CVID patients. In any event, and regardless of its origin, the immunosuppressive character of IVIg-induced monocytic MDSC might help in protecting the host from the extensive tissue damage caused by the excessive monocyte activation usually observed in CVID [73]. Moreover, and since MDSC also appear to increase immune surveillance and innate immune responses [79], IVIg-induced MDSC might also contribute to maintain immune homeostasis and improve antimicrobial activities in CVID patients. Of note, we have not seen any differences in IVIg-induced MDSC increases between patients with distinct duration of IVIg treatment. However, considering the limited cohort we have analyzed, future studies should assess prospectively whether the duration of IVIg treatment has any effect on the IVIg-induced changes in MDSC population that we now report.

Regarding the IVIg-mediated decrease in the intermediate and non-classical monocyte subsets in CVID, seen at the phenotypic and transcriptional levels, our findings support the idea that IVIg can correct the imbalance of monocytes subsets seen in CVID patients, which exhibit increased levels of CD16^+^ monocytes [54, 73]. Indeed, our results corroborate previous findings on the ability of IVIg to diminish the number of non-classical monocytes in after 4 h in CVID patients [54, 80–82], an effect that appears to be transient [80] and has been also observed in patients with Kawasaki disease [83]. Therefore, considering that CD16^+^ monocytes exhibit more pro-inflammatory ability than classical monocytes [50] and give rise to macrophages with a more pro-inflammatory gene profile [84], the expression of genes preferentially expressed in intermediate/non-classical monocytes (Fig. 2D) could be used as molecular markers for immediate/early responses to IVIg infusion.

An additional consequence of the IVIg-mediated M-MDSC increase in CVID patients is its potential involvement in the generation of regulatory T lymphocytes (T_reg_). Numerous reports have now established that IVIg enhances suppressive T_reg_ [85–88], an effect observed in immune thrombocytopenia [89], Guillain-Barré syndrome [90], Myasthenia Gravis [91], allergic airways disease [92], Kawasaki disease [93], and experimental autoimmune encephalomyelitis [94]. In fact, T_reg_ expansion has been proposed as a biomarker to predict clinical response to IVIg therapy [95], and is thought to be one of the mechanisms by which IVIg restores homeostasis in patients with autoimmune and systemic inflammatory disorders. Since MDSC promote T_reg_ expansion [96–100] and recruitment [101], the IVIg-induced increase in peripheral blood MDSC that we have observed in CVID has additional implications, and might be a primary step in the immunosuppressive ability of IVIg. While the global IVIg-induced anti-inflammatory effects may be beneficial to restrain chronic immune activation and inflammation in the complex interplay of factors involved in oncogenesis in CVID population [102], a major concern of our data is the potential deleterious effects of IVIg on patients with established cancer. This latter aspect deserves further focused exploration.

In summary, we report that IVIg infusion has an immediate effect on the transcriptome, phenotype and function of peripheral blood monocytes in CVID patients, and that these IVIg-induced changes are compatible with IVIg promoting the acquisition of M-MDSC-like properties upon infusion. These results warrant further analysis of potential similar IVIg effects in other diseases, especially considering the ample immunomodulatory actions of MDSC and the large number of disorders that are currently treated with IVIg.

## Supplementary Information

Below is the link to the electronic supplementary material.

Supplementary file1 (PDF 591 KB)

## Data Availability

The datasets generated during the current study were deposited in the Gene Expression Omnibus (http://www.ncbi.nlm.nih.gov/geo/) under accession no. GSE133835 (PBMCs), GSE133907 (CD14 cells), GSE158576 (CD20 cells), GSE158573 (CD3 cells).
