# Supplementary material for: Intravenous Immunoglobulins Promote an Expansion of Monocytic Myeloid-Derived Suppressor Cells (MDSC) in CVID Patients
Source: J Clin Immunol. 2022 Apr 29;42(5):1093–105. doi: 10.1007/s10875-022-01277-7 (PMC9053130; doi:10.1007/s10875-022-01277-7)
Supplement: Supplementary file 1 — Supplementary file1 (PDF 591 KB) [file 10875_2022_1277_MOESM1_ESM.pdf]

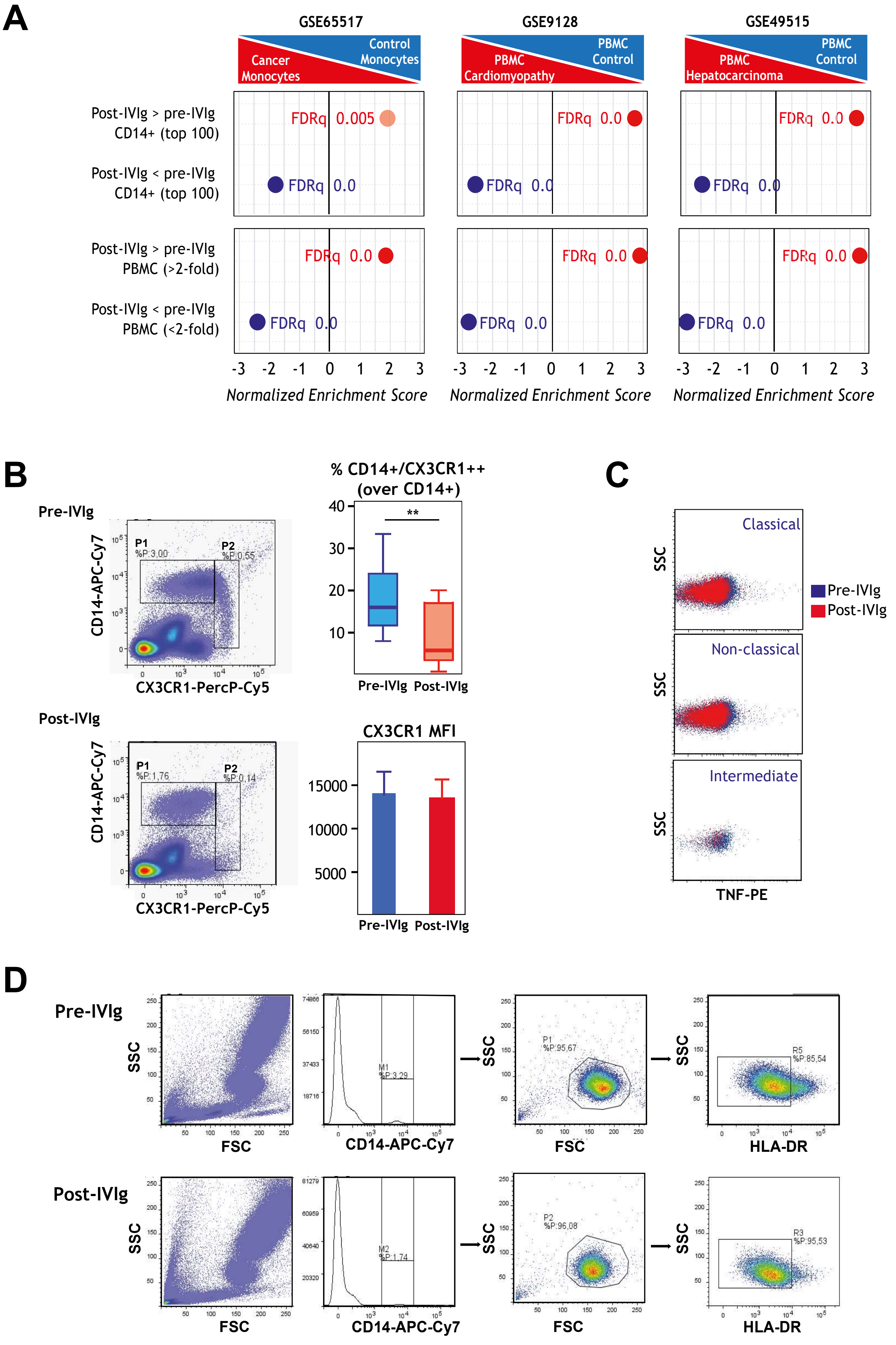

Supplementary Figure 1

Supplementary Figure 1. A. The expression of IVIg-regulated genes is skewed in PBMC from pathologies exhibiting altered myeloid differentiation. Raw data from GSEA with the gene sets containing the genes Upregulated [”Post-IVIg > pre-IVIg CD14$^+$ (top 100)”] or Downregulated [”Post-IVIg < pre-IVIg CD14$^+$ (top 100)”] in CD14$^+$ post-IVIg relative to CD14$^+$ pre-IVIg (upper panels), and the genes Upregulated [”Post-IVIg > pre-IVIg PBMC (>2-fold)”] or Downregulated [”Post-IVIg < pre-IVIg PBMC (<2-fold)”] in PBMC post-IVIg relative to PBMC pre-IVIg (lower panels), on the ranked comparison of the indicated transcriptomes, retrieved from from GEO GSE65517, GSE9128 and GSE49515. In all cases, Normalized Enrichment Score (NES) and FDRq is indicated. **B.** Phenotypic evidences for the IVIg-mediated alteration of the proportion of monocyte subsets in peripheral blood from CVID patients. CX3CR1 expression on the cell surface of monocytes before and after IVIg treatment in CVID patients. Monocytes were gated by CD14 and CX3CR1 expression. The percentage of CX3CR1^{++}/CD14$^+$ cells in all CD14+ monocytes and CX3CR1 mean expression for CX3CR1^{++}/CD14$^+$ monocytes are shown. Mean±SEM of 8 patients are shown (**, p<0.01). **C.** Determination of TNF expression in the three monocyte subsets (Classical, Intermediate, Non-classical) in CVID patients before (pre-IVIg) and after IVIg administration (post-IVIg), as determined by flow cytometry. A representative flow cytometry profile from a single CVID patient is shown. **D.** Peripheral blood monocytes from IVIg-treated CVID patients display MDSC-like phenotype. The gating strategy for the identification of HLAlow monocytes in peripheral blood from CVID patients before (pre-IVIg) and after IVIg infusion (Post-IVIg) is shown. First, monocytes were selected by high expression of CD14 and then, gated by FSC and SSC parameters. Selected cells were later analyzed by HLA expression.

Supplementary Table 1. Patients' demographic distribution, clinical characteristics and classification according to CVID clinical phenotypes. EBV: Epstein-Barr virus; GLILD: granulomatous–lymphocytic interstitial lung disease; ITP: immune thrombocytopenia; TB: tuberculosis; UTIs: urinary tract infections.

Patient No.	Age (yrs)	Gender	Clinical Manifestations	CVID clinical phenotype.*	Duration of IVIg treatment (yrs)
#1	40-45	M	Complicated pneumonia, sinusitis, mild bronchiectasis, Helicobacter pylori gastritis, external otitis.	**No-disease related complications** (only infections).	5
#2	20-25	F	Uncomplicated pneumonia, acute sinusitis, uncomplicated EBV viremia.	**No-disease related complications** (only infections).	5
#3	55-60	F	Recurrent upper tract infections (sinusitis with surgical pneumatization on 2 occasions) unexplained enteropathy with chronic diarrhea, lymphoid lymphoproliferation, suppurated internal otitis, recurrent UTIs, refractory H pylori infection.	**Polyclonal lymphocytic infiltration plus unexplained enteropathy.**	14
#4	55-60	F	ITP requiring splenomegaly, Helicobacter pylori infection, unexplained enteropathy (nonspecific colitis) with lymphoid follicular hyperplasia, recurrent otitis, recurrent sinusitis and bronchitis with atelectasis and bronchiectasis with lobectomy.	**Cytopenia, polyclonal lymphocytic infiltration and unexplained enteropathy.**	**7**
#5	30-35	F	Uncomplicated EBV viremia, recurrent upper and lower respiratory infections, recurrent conjunctivitis, bronchiectasis.	**No-disease related complications** (only infections).	6
#6	65-70	F	Hepatitis A, typhoid fever and TB in childhood. Lymphoid granulomatosis with pulmonary interstitial involvement, chronic pigmentary purpuric dermatosis, papillary mucinous intraductal tumor of the head of the pancreas, micronodular splenomegaly suggestive of a lymphoproliferative process.	**Polyclonal lymphocytic infiltration.**	6
#7	55-60	M	Recurrent lower respiratory infections, Uncomplicated pneumonia. Herpes zoster.	**No-disease related complications** (only infections).	4
#8	55-60	M	Malignancy (non-Hodgkin lymphoma), Recurrent oral ulcers, chronic lymphatic leukemia uncomplicated pneumonia, arthralgia.	**Polyclonal lymphocytic infiltration.**	3
#9	55-60	F	Recurrent respiratory and gastrointestinal infections.	**No-disease related complications** (only infections).	7
#10	35-39	F	Severe GLILD, viral hepatitis responding to treatment, symptomatic splenomegaly, mild asymptomatic cytopenia.	**Polyclonal lymphocytic infiltration plus cytopenia.**	7
#11	40-45	F	Arthritis, cytopenias, multiple antibiotic allergies, acute sinusitis, asymptomatic increase of liver enzymes.	**Cytopenias.**	49

* Classification according to H. Chapel, M. Lucas, S. Patel, M. Lee, C. Cunningham-Rundles, E. Resnick, L. Gerard, E. Oksenhendler, Confirmation and improvement of criteria for clinical phenotyping in common variable immunodeficiency disorders in replicate cohorts, J. Allergy Clin. Immunol. 130 (2012) 1197–1198, https://doi.org/10.1016/j.jaci.2012.05.046 e9.
